# Underrepresented Populations at the Archaic Introgression Frontier

**DOI:** 10.3389/fgene.2022.821170

**Published:** 2022-02-25

**Authors:** Fernando A Villanea, Kelsey E. Witt

**Affiliations:** ^1^ Department of Anthropology, College of Arts and Sciences, University of Colorado Boulder, Boulder, CO, United States; ^2^ Department of Ecology and Evolutionary Biology, Brown University, Providence, RI, United States

**Keywords:** ethics, underrepresented populations, archaic admixture, anthropology, human genomics

## Introduction

Recent advancements in the recovery of ancient genomes have yielded high-coverage sequences for two archaic human species: Neanderthals and Denisovans. Perhaps more surprisingly, direct comparisons of archaic and modern human genomes have revealed a complex landscape of admixture between both archaic species and modern humans (Browning et al., 2018; Villanea and Schraiber, 2019). While we call these regions of the human genome “archaically introgressed”, they are functional contributors to the living human gene pool, affecting our health and fitness. For Neanderthals in particular, early archaic ancestry maps focused on modern Eurasians, as hundreds of genomes from Europe and East Asia were readily available from the 1000 Genomes Project (see Sankararaman et al., 2014; Vernot and Akey, 2014). Coupled with the geographic distribution of Neanderthal archaeological sites, which are largely located in Europe, this created a strong impression to the larger public that individuals of European descent, in particular, carried archaic genomic elements, which coincided with a larger interest in commodifying archaic ancestry by personalized genomic companies—as evidenced by 23 and Me incorporating a report of Neanderthal ancestry into their mainline product.

**FIGURE 1:**
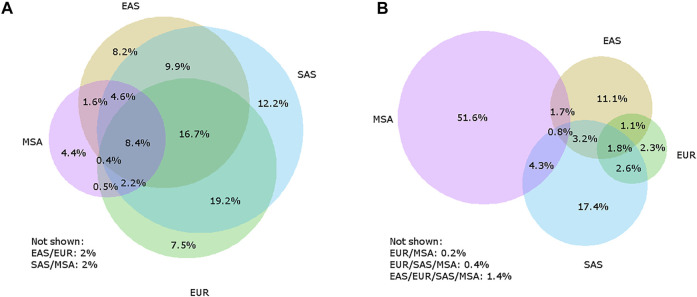
Venn diagrams illustrating how archaic variants are shared between Europeans (EUR), East Asians (EAS), and South Asians (SAS) as defined by the 1000 Genomes Project, and Maritime Southeast Asians (MSA) from the Simons Genome Diversity Project for Neanderthal-unique **(A)** and Denisovan-unique **(B)** variants. The listed percentages are the percent of the total number of Neanderthal-unique or Denisovan-unique variants. It is not always possible to accurately display all possible overlaps with a four-circle Venn diagram, so any sets that are not shown have percentages included below each Venn diagram, with the circles overlapping listed. For example, “EAS/EUR” refers to all variants that are shared between East Asians and Europeans but are not found in South Asians or Maritime Southeast Asians.

As scientific efforts progressed to identify regions of the modern human genome that originated in these archaic populations, in a twist of irony, European populations were found to retain the smallest component of both Neanderthal and Denisovan genome ancestry outside of African populations (Sankararaman et al., 2016). Current archaic genome studies indicate that South Asian populations, as defined by the 1000 Genomes Project (which encompasses populations from Bangladesh, India, Pakistan, and Sri Lanka), have a larger component of Neanderthal ancestry than Europeans, East Asians, or Maritime Southeast Asians (Witt et al., 2021 bioRXiv, Figure 1). Conversely, Maritime Southeast Asian populations retain a larger component of the genome, and more unique variation, of Denisovan ancestry (Sankararaman et al., 2016; Vernot et al., 2016, Figure 1). As studies continued to identify regions of the human genome enriched for archaic ancestry at the population level, a consistent pattern emerged for the distribution of archaic ancestry: for a vast majority of genes found in modern humans, archaic variants appear to have been removed from the gene pool by negative natural selection (Harris and Nielson, 2016; Petr et al., 2019), but there is also enrichment in a minority of functional regions that indicate positive selection (Racimo et al., 2017; Jagoda et al., 2018). Thus, the focus has shifted from viewing archaic ancestry as a quirk of human evolution into understanding the functional importance of these rare genomic regions enriched for archaic ancestry.

## Functional Importance of Archaic Ancestry

There are multiple examples of archaic gene variants that helped modern humans to adapt to novel environments as they expanded throughout the world, such as differences in UV radiation exposure, temperature, dietary composition, and pathogen exposure. Functional genes enriched for archaic ancestry include the Neanderthal BNC2 and OCA2 variants related to skin pigmentation (Sankararaman et al., 2014; Vernot and Akey 2015; Gittelman et al., 2016); the OAS locus and Toll-like receptor loci related to immune response (Mendez et al., 2012; Dannemann and Kelso 2016; Gittelman et al., 2016; Sams et al., 2016); and the TBX15/WARS2 locus related to lipid metabolism (Racimo et al., 2017). Furthermore, there is mounting evidence that the bulk of contributions to modern human fitness and health from archaic ancestry is through non-gene functional portions of the human genome, which are far more difficult to conceptualize (Dannemann et al., 2017; Petr et al., 2019; Silvert et al., 2019). For example, some enhancer regions show enrichment in Neanderthal alleles, such as adipose-related tissues and primary T-cells (Dannemann et al., 2017; Petr et al., 2019; Silvert et al., 2019), plus, up to an additional forty-two tissues in humans show significant enrichment of archaic alleles in enhancers, with the highest rate of enrichment identified in adipose-related tissues and immune cells (Silvert et al., 2019).

Adaptive introgression seems particularly prominent in immune-related functional genome elements, suggesting that Neanderthals and Denisovans harbored many alleles adapted to local pathogens that were positively selected after introgression into modern humans (Ahlquist et al., 2021). Genes that encode proteins that interact with RNA viruses are also enriched for introgressed alleles (Enard and Petrov, 2018). Similarly, polygenic adaptive introgression has been reported in pathways associated with immunity (Gouy and Excoffier 2020). Finally, population transcriptome studies of immune response to viral and bacterial pathogens have found many gene expression and splicing differences between individuals of European and African ancestry that appear to be driven by Neanderthal introgressed alleles, providing further support for their regulatory impact on immunity (Nedelec et al., 2016; Quach et al., 2016; Rotival et al., 2019). Recently, a study reporting the expression of Neanderthal non-gene elements found as many as 292 expression-modulating variants in human lineages, most of them related to immune function, underlining the importance of archaic variation in modulating the expression of modern human genes (Jagoda et al., 2021). Although the archaic populations that admixed with modern humans are now extinct, the archaic variation remaining in the human gene pool can have significant impacts on health, especially immune function, and therefore is an important target for genomics and biomedical research.

## Importance of Non-European Genomes for Understanding Archaic Genetic Variation

The field of genomics has a well-known Eurocentric bias, where European populations are more broadly sampled and variation found in European populations is far better characterized than other populations (e.g. Popejoy and Fullerton, 2016). Interestingly, despite this thorough sampling, a larger potential for discovering novel adaptive archaic introgression exists in non-European genomes, as Europeans have some of the lowest proportions of both Neanderthal and Denisovan ancestry outside of African populations (Sankararaman et al., 2014, 2016).

Given the recent interest in identifying archaic functional variants in modern humans, a more effective study design is to examine diverse populations that have thrived in varied environmental conditions, to identify archaic variants that may have been adaptive. For example, the most compelling case of adaptive introgression of an archaic gene to date is the high-altitude adaptation of Tibetans achieved through selection for the archaic variant of the EPAS1 gene, which was introduced into the ancestral Tibetan populations through admixture with Denisovans (Huerta-Sánchez et al., 2014; Zhang et al., 2021). This example highlights how genetic variation unique to Tibetans was paramount in understanding the role of archaic variation in modern humans in the first place, variation that simply does not exist in any other population. Another example of an adaptive archaic variant found in underrepresented populations is a Denisovan variant of TBX15, which is found at high frequency in Greenlandic Inuit (Racimo et al., 2018). This Denisovan haplotype is found in many Indigenous populations of the Americas at a high frequency (>0.8), and in Inuit specifically has a strong signal of positive selection. TBX15 is linked to a number of phenotypes, including lipid metabolism, especially at cold temperatures, which suggests that the Denisovan variant may have been adaptive for humans as they populated the Arctic. The archaic alleles are also in linkage disequilibrium with a SNP that is linked to waist-hip ratio, with an effect size of 0.034 (Heid et al., 2010; Fumagalli et al., 2015). This SNP was identified in European populations, but the haplotype in Greenlandic Inuit is not well-characterizing, underlining the need for further study of these underrepresented populations. These two examples suggest that the further study of populations that are generally underrepresented in genomic research, including Melanesians, Southeast Asians, and Indigenous American populations, could yield additional novel archaic functional variants.

While many of the early genetic analyses of archaic ancestry in modern humans have focused on continental Eurasian populations (especially Europeans and East Asians), Papuans and other populations in Oceania have a unique distribution of archaic ancestry (Figure 1). Papuans have a high proportion of Neanderthal ancestry, but most notably they have an extremely high proportion of Denisovan ancestry compared to other populations worldwide - in some populations, as high as 5% (Sankararaman et al., 2016; Vernot et al., 2016). Additionally, evidence suggests that Papuans have Denisovan ancestry from two genetically distinct Denisovan populations (Browning et al., 2018; Jacobs et al., 2019). This is evident as the majority of Denisovan variants in Papuans are not shared with Eurasian populations (Figure 1). Recently, the Ayta Magbukon people in the Philippines have been reported to possess Denisovan ancestry proportions even higher than that of Papuans (Larena et al., 2021). Southeast Asia also has a long history of occupation by multiple archaic hominins, including *Homo erectus* (Rizal et al., 2020) and *Homo floresiensis* (Brown et al., 2004; Sutikna et al., 2016)*,* and possibly a new hominin, currently named *Homo luzonensis* (Detroit et al., 2019). Some of these hominins may have lived in the region around the same time period, and some likely overlapped with humans although evidence for gene flow between these hominins and modern humans has yet to be identified (Teixeira et al., 2021).

Another underrepresented group of populations with an interesting legacy of archaic ancestry are Indigenous Americans. Indigenous American populations today are the descendants of individuals who populated the American continent in a process that started at least 25,000 years before present (Bennet et al., 2021). Indigenous American individuals carry archaic genomic elements at frequencies comparable to modern East Asian individuals (Sankararaman et al., 2016), indicating that the first American migrants already carried archaic genomic elements. In the 25,000 or more years since these founding populations migrated to the Americas, these peoples would have encountered numerous novel environments, and adapting to meet those environmental challenges helped to shape their genomes into the unique populations living today. This long adaptation process would have undoubtedly also impacted the distribution of archaic genomic elements in Indigenous American populations.

**FIGURE 2:**
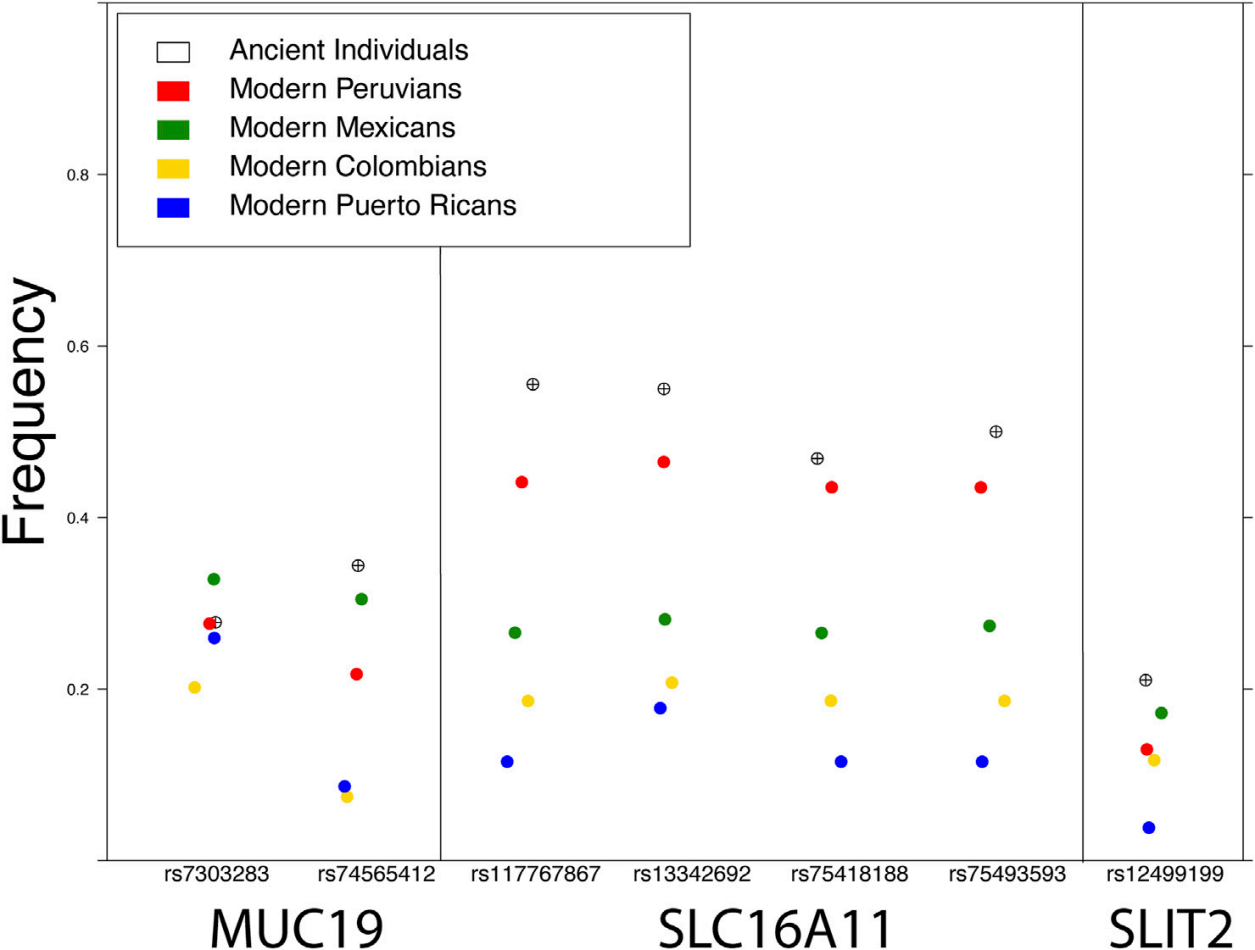
Frequency of the archaic variant of SNPs in three genes reported under selection in modern Indigenous American populations: MUC19 (Racimo et al., 2016; Reynolds et al., 2019), SLC16A11 (Williams et al., 2014), and SLIT2 (Reynolds et al., 2019). The frequency in ancient individuals was calculated from various pre-European colonization genomes available in the literature (see Figure *Methods*).

The European colonization of much of the American continent has also left a profound impact on its inhabitants, visible in the genomes of all individuals today, as most modern Indigenous American individuals have both European ancestry as a result of colonization and African ancestry as a result of African individuals being forcibly relocated through the slave trade. This admixture has also affected the amount of archaic ancestry in modern Indigenous American populations: admixture with Europeans and Africans, which have slightly less and significantly less archaic ancestry respectively, resulted in a dilution of archaic ancestry in modern Indigenous American descendants (Figure 2). Natural selection has been an important force in shaping Indigenous American genomes, both pre-admixture (Williams et al., 2014; Reynolds et al., 2016) as well as post-admixture (Ongaro et al., 2021). Recent admixture between populations can have both positive and negative health consequences: for example, individuals with recent ancestors from multiple worldwide populations show reduced risk for some genetic disorders but an increased incidence of autoimmune disease (Rudan, 2006; Martin et al., 2017). Therefore, the dilution of archaic elements in modern Indigenous populations could provide health benefits, but could also have a negative impact on health: by replacement with maladaptive gene variants, by breaking up existing epistatic gene interactions, or by interactions between gene expression-modulating variants. Because of these changes to genetic architecture as a result of admixture following European contact, advances in personalized medicine for Indigenous American individuals could be extremely beneficial, yet any future endeavor requires addressing a long history of bad faith interactions between geneticists and Indigenous American communities.

While it is clear that the study of archaic introgression in modern humans would benefit from the analysis of populations that are often underrepresented in genomic research, a balance must be struck between the scientific desire for knowledge and respect for these populations and their right to control their own data. Past work by geneticists with marginalized communities has often been exploitative, involved little interaction between researchers and communities after samples were collected, and rarely resulted in benefits for the individuals being studied (e.g., Hart and Sobraske, 2003). This history of poor interactions between the scientific community and marginalized populations has resulted in the erosion of trust in scientists, and some communities have rightfully placed a moratorium on genetic research to limit the infliction of future harms (Begay et al., 2020; Claw et al., 2021). Many Indigenous scholars have provided suggestions and guidelines to ensure that future genomic research is conducted in an equitable way that ensures communities are able to make their own decisions concerning their research and data, and also equipping them to conduct the research themselves (Claw et al., 2018; Fox, 2020; Hudson et al., 2020; Tsosie et al., 2020).

Community organization is key for maintaining data sovereignty and creating leverage for the distribution of material benefits arising from unique genomic variation. The Khoisan communities of South Africa have published the San Code of Research Ethics (globalcodeofconduct.org/affiliated-codes/), in which they collectively dictate how they will interact with researchers. Global measures implemented to help ensure communities being studied benefit from the material returns of genetic research include the “Nagoya Protocol on Access and Benefit-sharing” (cbd.int/abs/); an international agreement that established guidelines for “sharing the benefits arising from the utilization of genetic resources in a fair and equitable way”, and as of November 2020 the agreement has been ratified or accessioned by 132 countries. Other communities have also chosen to control their own genetic resources. For example, the Native BioData Consortium (NBDC, https://nativebio.org/), was founded in 2018 by Indigenous scientists as a biobank for Indigenous communities. The NBDC also conducts its own research projects and hosts training workshops for Indigenous students. Further examples of community interests built into genomic projects include initiatives such as Variant Bio (variantbio.com/), a company which forms partnerships with communities possessing unique genetic diversity, builds the priorities of the communities into their study designs, and is committed to redistributing royalties from any medically important discoveries originating from their genomes.

In the near future, research into further evolutionary adaptations born of archaic genome elements will drive the need to genotype other underrepresented populations globally. As the funding available for these studies is largely Eurocentric, but the genetic diversity of interest exists outside of Europeans, this may result in exploitative research designs. Because of this asymmetry, it is paramount to build scientific capacity within these communities, and prioritize funding for existing scientists of underrepresented populations. At the same time, community engagement needs to be built into all levels of study designs; research and funding schemes must be intrinsically flexible to accommodate the priorities of the communities co-designing the studies.

Likewise, ultimate control of data sharing should be managed directly by community-lead organizations. While open science has been championed as an ideal model, the current application of open science has neither benefited Indigenous communities, nor is it particularly “open” as it is currently implemented. As an example, publicly available human genomic data is already compartmentalized between repositories such as the 1000 Genomes Project, the Simons Genome Diversity Project, and the Human Genome Diversity Project, among other more specialized databases, in addition to research groups that host and maintain their own data, such as the Neanderthal and Denisovan genomes from the Max Planck Institute for Evolutionary Anthropology. Each one of these entities determines what permissions are required to access their data, meaning that in practice, there is not one centralized way to access human genomic data. Going forward, the addition of community-led data management would maintain similar compartmentalization as exists today, but provide Indigenous communities with leverage in negotiations on how that data is used (Mc Cartney et al., 2022). As legal protections and community engagement procedures are more well-established in some regions of the world than others, the community-involved research should be conducted not just with respect to local laws, but in a way that truly puts decision-making in the hands of the populations being studied. Finally, the format for the dissemination of results should recognize the communication traditions of participating communities to maximize digestibility of scientific and medical findings, to ensure transparency and informed consent during every study step. This transparency in reporting should also explicitly inform the financial potential for practical applications of new findings, to provide leverage for the negotiations of royalties from any medically important discoveries stemming from their unique genomic ancestry.

## Figure Methods

Figure 1 To identify sites containing archaic alleles, we used a method previously employed by Witt et al. (2021, biorXiv) that considers archaic alleles to be shared with modern humans and Neanderthals/Denisovans, present in African populations at a frequency of less than 1%, and present in a population outside of Africa with a frequency of at least 1%. We used the 1000 Genomes Project (1KGP) genomic data for our African comparison population and identified archaic alleles in the non-African 1KGP populations as well as Papuans, whose genomes were collected as part of the Simons Genome Diversity Project (SGDP). We classified those populations into regions (Americas, Europe, East Asia, South Asia, and Papua New Guinea, as defined by the 1KGP and SGDP, and determined which positions in the genome contained archaic alleles for each region. To examine the patterns of variant sharing between Neanderthals and Denisovans separately, we only considered variants present in one population and absent from the other, which we term Neanderthal-unique and Denisovan-unique variants.

Figure 2 Modern frequencies were calculated from four populations publicly available in the 1000 Genomes Project. Frequencies in ancient individuals were calculated by combining high coverage (>1X) pre-European contact genomes from the literature, including nine individuals from California and one from Ontario (Scheib et al., 2018), four from Peru (Lindo et al., 2018), four from Patagonia (de la Fuente et al., 2018), one from Alaska (Moreno-Mayar et al., 2018), and one from Montana (Rasmussen et al., 2014).
